# Reconfigurable electro-optical logic gates using a 2-layer multilayer perceptron

**DOI:** 10.1038/s41598-022-18408-0

**Published:** 2022-08-20

**Authors:** Chu-En Lin, Yueh-Heng Lu, Meng-Ting Zhou, Chii-Chang Chen

**Affiliations:** 1 Department of Electronic Engineering, National Chin-Yi University of Technology, Taichung, 411030 Taiwan; 2 Department of Optics and Photonics, National Central University, Zhongli, 320317 Taiwan

**Keywords:** Information theory and computation, Integrated optics

## Abstract

In this work, we aim to use the optical amplifiers, directional couplers and phase modulators to build the electro-optical gates. Thanks to the 2-layer-multilayer-perceptron structure, the inversion of matrix is performed to obtain the coupling ratio of the directional couplers and the phase delay of the phase modulators. The electro-optical OR, AND, XOR, NAND, NOR and XNOR gates are demonstrated. Moreover, we not only study the results under the ideal condition of device, but also discuss the imperfect situation with 1% error of fabrication or operation to study the tolerance of this system. Through our simulation results, the visibility of the gate output can be higher than 0.83. The gates can be fabricated in a silicon-based chip to develop the integrated optics computing system.

## Introduction

Optical computing is a striking issue in recent years. The logic gates are the fundamental devices to carry out this task^1^. All-optical logic gates based on photonic crystal (PC) waveguides, and ring resonators were proposed recently^2–4^. Also, there are many all-optical structures proposed to demonstrate the all-optical reservoir computing devices^5–8^. Thanks to the progress of the photonic technology which have been investigated intensively in recent decades, we can make these devices as a tiny chip^9–12^. Moreover, the temporal solitons and the continuous-wave light sources were used as the carriers of the input and output signals. The refractive index change of the waveguide by means of non-linear Kerr effect with high power light beam produces the tuning or switching of the output states of logic gates^13,14^. However, a long propagation path around several micrometers or even millimeters through the dielectric waveguides to deliver the light from light source to the logic gates might not be avoidable in a dielectric material (such as silicon) chip. The light might be absorbed by the waveguides leading to the deformation of the solitons. The low absorption and dispersion waveguides such as the hollow waveguide or using 3-dimensional PC structure over the waveguide might be considered to connect the light sources and the logic gates as well as to connect between the logic gates^15–17^.

Active components such as a tri-mode laser, Fabry–Perot laser, and semiconductor saturable absorber had also been proposed to form the all-optical logic gates^14,18,19^. Through tuning the optical injection of the tri-mode lasers, the change of logical state can be achieved^14^. High-speed all-optical logic gates at the bit rate of 340 Gb/s using the cross-gain modulation in semiconductor optical amplifier has also been achieved^19^.

Recently, researchers proposed the logic gates which are formed by microring/microdisk modulator^20,21^. Although the microring-/microdisk-modulators are tinier than our logic gate, the control process is more complicated than our design. With regard to the microring-modulator logic gates, they can achieve only AND, OR and XOR gates^20^. However, they need to modify the optical structure to carry out the corresponding function. In our study, we only need to tune the coupling ratio of the directional couplers and the phase of the phase shifter to achieve 6 logic gates at the single output port. For microdisk-modulator logic gates, the authors also can achieve OR, NOR, XNOR, XOR, AND and NAND logic gates^21^. However, the tunable laser providing 3 different wavelengths should be used. The size of the laser source and the external cavity to tune the wavelength might occupy several hundred square micrometers or even several square millimeters. The dimension of the integrated chip could be similar to that of our design.

In our previous work, we took advantage of the reservoir computing (RC) and unsupervised learning method optical neuron networks to establish an XOR gate^5^. This XOR gate with low bit error ratio was obtained by scanning the delay of optical phase shifter. In the present work, an electro-optical (EO) logic gates based on RC without the recurrent property are proposed. Obviously, it is a 2-layer-multilayer-perceptron (2-layer MLP) structure. This logic gates are composed of the optical amplifiers, directional couplers (DCs), and phase modulators (PMs). Comparing this new logic gates to our previous works, the feedback signal (the recurrent property) is removed in this new design. The switching time between 0 and 1 for the output of the gates can be shortened. In this work, the matrix inversion is performed to calculate the coupling ratio of the DCs and the phase delay of the PMs using the target output of the logic gates in the same optical configuration. The reconfigurable electro-optical OR, AND, XOR, NAND, NOR and XNOR gates formed by one optical structure are demonstrated.

## Design of the 2-layer-multilayer-perceptron structure

It is known that RC system has been extensively used in the development of logic gates, waveform recognition, and rainfall prediction^5,22–24^. This system is composed of the input weight matrix ${W}^{in}$, the recurrent weight matrix $W$, and the readout matrix ${W}^{out}$. The input matrix ${W}^{in}$ is used to scale the size of the input data to the size of the matrix *W*. The value of the elements in *W*^*in*^ and *W* is chosen randomly. In this study, the learning is completed in a single pass-through training data. The optimal readout matrix ${W}^{out}$ is used to generate the output of the RC system.

**Figure 1:**
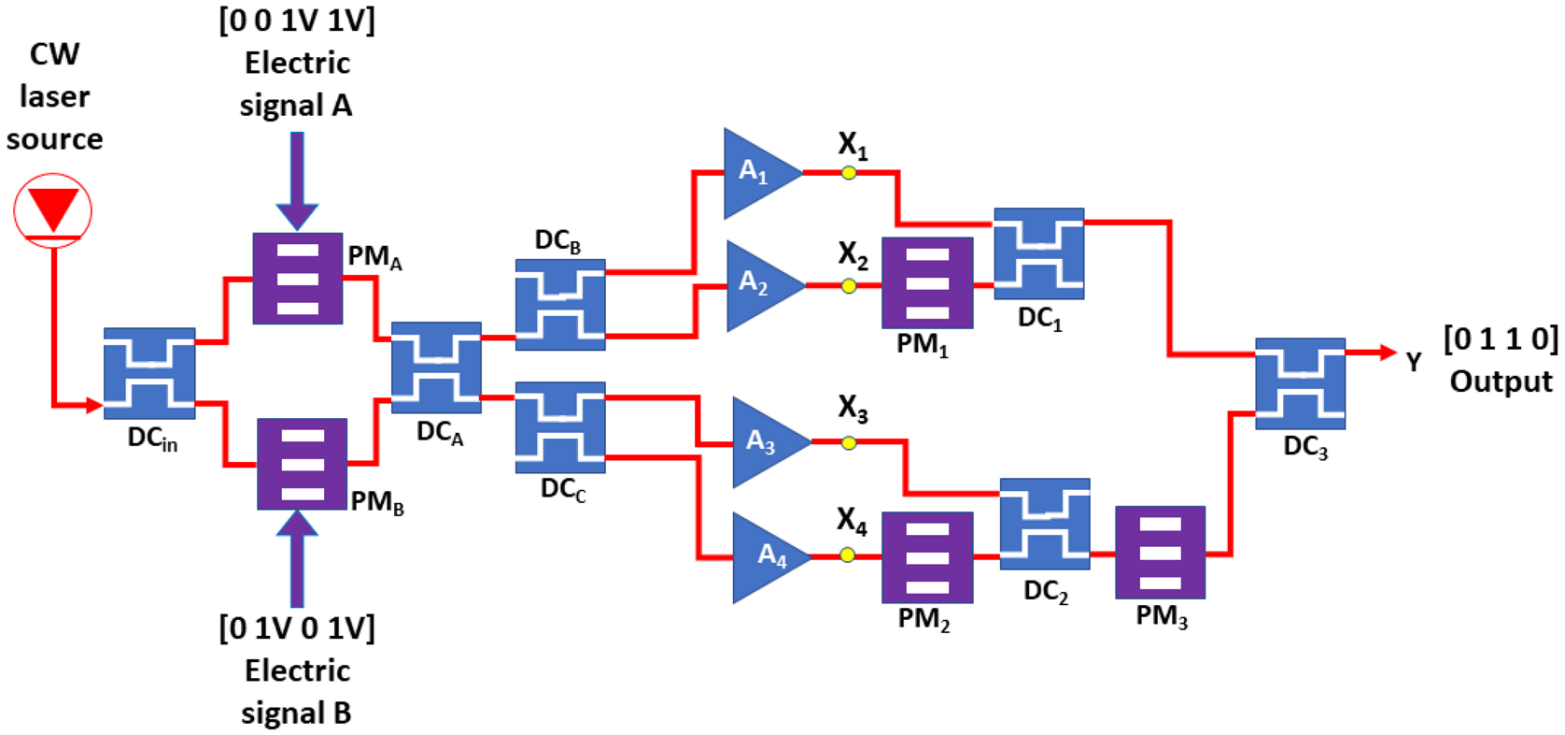
The optical setup of this EO logic gate. CW, DC and PM stand for the continuous wave, the 2 by 2 directional coupler, and the phase modulator. The possible recombination of the input signals A and B is (0, 0), (0, 1 V), (1 V, 0) and (1 V, 1 V). The corresponding *Y*^*target*^ for the XOR gate is [0 1 1 0].

In RC system, the neuron can be described as a function of the current input and its previous calculation result which can be expressed by^25^

1 $$X\left(n\right)=\left(1-L\right)X\left(n-1\right) +Lf\left({{\varvec{W}}}^{in}u\left(n\right)+{\varvec{W}}X(n-1)\right)$$

where the function *f* is the nonlinear activation function of the neuron. Usually, the hyperbolic tangent function, tanh(), is used as the nonlinear activation function to converge the output value of neuron within − 1 and 1^26^. In this research, the non-linear function of our optical neuron is provided by the optical amplifiers. (The optical setup is shown in Fig. 1.) $L$ is the leaky rate which is a real number from 0 to 1. However, since we do not use the recurrent property of RC in the optical neural network, *L* and *W* are set to be 1 and null, respectively producing a 2-layer MLP structure. $u\left(n\right)$ represents the *n*-th input data. Thus, the reservoir activation state, $X\left(n\right)$, is modified to $f\left({{\varvec{W}}}^{in}u\left(n\right)\right)$. The network target output ${Y}^{target}$ is given by

2 $${Y}^{target}={W}^{out}X$$

3 $${W}^{out}=\left[\begin{array}{ccc}{w}_{1}& {w}_{2}& \begin{array}{cc}\dots & {w}_{M}\end{array}\end{array}\right]$$

4 $$X=\left[\begin{array}{c}\begin{array}{c}{X}_{1}(1)\\ {X}_{2}(1)\\ \dots \end{array}\\ {X}_{M}(1)\end{array} \begin{array}{c}\begin{array}{c}{X}_{1}(2)\\ {X}_{2}(2)\\ \dots \end{array}\\ {X}_{M}(2)\end{array}\dots \begin{array}{c}\begin{array}{c}{X}_{1}(N)\\ {X}_{2}(N)\\ \dots \end{array}\\ {X}_{M}(N)\end{array}\right]$$

In Eq. (3), *M* and *N* are the number of the neurons and the number of the input data, respectively. By collecting the training data $X$ and the target training signals ${Y}^{target}$, the readout matrix ${W}^{out}$ can be obtained by inversing the matrix *X* from Eq. (2).

5 $${W}^{out}={Y}^{target}{X}^{-1}$$

The main feature of 2-layer MLP is the fact that the training (or the optimization) is done by dealing with the readout matrix ${W}^{out}$ using the inverse of the matrix *X*. As we mentioned above, once we obtain the elements of ${W}^{out}$, we could deduce the coupling ratio and phase in our DCs and PMs, respectively. This will be detailed in the next section. Through this method, we can obtain the parameters of the optical devices in this optical neural network.

## Methods

Figure 1 illustrates the optical setup of our EO neural-network-based logic gates. The CW laser source at the wavelength of 1550 nm with the power of 0.1 W is launched into the DC_in_ of our logic gate. The optical nonlinear effect of the optical fibers which connect the optical devices are ignored. This DC_in_ with the coupling ratio of 50% is used to divide equally the input light into two light beams. The two light beams are launched into the two phase modulators (PM_A_ and PM_B_ in Fig. 1) individually. The half-wave voltage V_π_ of the PM_A_ and PM_B_ is set randomly to be 1 V and 2 V, respectively. The input signals A and B are applied on the phase modulators PM_A_ and PM_B_, respectively. The applied voltages are 0 or 1 V of the phase modulators to present the logic 0 and 1, respectively. PM_A_ and PM_B_ are connected to the directional coupler DC_A_. After that, the output signals of DC_A_ are launched into DC_B_ and DC_C_. DC_A_, DC_B_ and DC_C_ serve as the input matrix ***W***^*in*^ of the 2-layer MLP in which the values of the elements are chosen randomly. The coupling ratio of DC_A_, DC_B_ and DC_C_ is therefore set randomly. In our research, for the example of the XOR gate, the coupling ratios of the DC_A_, DC_B_ and DC_C_ are set randomly to be 0.56, 0.83 and 0.96, respectively.

The four outputs of the DC_B_ and DC_C_ are connected to the optical amplifiers A_1_, A_2_, A_3_ and A_4_, individually. The optical gain of the amplifiers is given by G_o_/(1 + G_0_P/P_sat_) where G_0_, P, P_sat_ are the small signal power gain, the power of the input light, and the saturation output power, respectively. For all amplifiers, the small signal power gain and the saturation output power are set to be 30 dB and 30dBm, respectively. The optical amplifiers provide the non-linear function which is required for 2-layer MLP system. The complex electric field of the output light of the amplifiers A_1_, A_2_, A_3_ and A_4_ are denoted by X_1_, X_2_, X_3_ and X_4_, respectively, which are regarded as the output of the neurons. Therefore, the number of the neurons, *M* in Eqs. (3) and (4) is 4. In other words, an optical amplifier can be regarded as an optical neuron. The readout matrix ${W}^{out}$ is composed of the 3 phase modulators PM_1_, PM_2_ and PM_3_ as well as the 3 directional couplers DC_1_, DC_2_ and DC_3_. ${\varnothing }_{1}$, ${\varnothing }_{2}$ and ${\varnothing }_{3}$ are the phase delay of the phase modulators PM_1_, PM_2_ and PM_3_, respectively. ${\alpha }_{1}$, ${\alpha }_{2}$ and ${\alpha }_{3}$ are the coupling ratios of the DC_1_, DC_2_ and DC_3_, respectively. The general formula of the output of electric field for the phase modulator and the directional coupler can be given by Eqs. (6) and (7), respectively.

6 $${X}_{out}={X}_{in}{e}^{i\varnothing }$$

7 $$\left[\begin{array}{c}{X}_{out1}\\ {X}_{out2}\end{array}\right]=\left[\begin{array}{cc}\sqrt{1-\alpha }& i\sqrt{\alpha }\\ i\sqrt{\alpha }& \sqrt{1-\alpha }\end{array}\right]\left[\begin{array}{c}{X}_{in1}\\ {X}_{in2}\end{array}\right]$$

where ${X}_{in}$ and ${X}_{out}$ represent the complex input and output electric fields of the components, respectively. $\varnothing$ and $\alpha$ are the phase delay of the phase modulators and the coupling ratio of the 2 by 2 DCs, respectively. The two input lights in the directional coupler are interfered. Using Eqs. (6) and (7), the complex electric field of the output light of the whole system ${Y}^{target}$ can be derived by

$${Y}^{target}=\sqrt{1-{\alpha }_{3}}\sqrt{1-{\alpha }_{1}}{X}_{1}+\mathrm{j}\sqrt{1-{\alpha }_{3}}\sqrt{{\alpha }_{1}}{e}^{i\left({\varnothing }_{1}\right)}{X}_{2}$$

8 $$+j\sqrt{{\alpha }_{3}}\sqrt{1-{\alpha }_{2}}{e}^{i({\varnothing }_{3})}{X}_{3}-\sqrt{{\alpha }_{3}}\sqrt{{\alpha }_{2}}{e}^{i({\varnothing }_{2}+{\varnothing }_{3})}{X}_{4}$$

The first step of the design process is to define the gate. For example, for an XOR gate, the possible recombination of the input signals A and B are (0, 0), (0, 1 V), (1 V, 0) and (1 V, 1 V). Thus, in Eq. (4), the number of the possible recombination of the input signals, $N$, is 4. The corresponding result ${Y}^{target}$ for the XOR gate are [0 1 1 0]. The complex electric fields X_1_, X_2_, X_3_ and X_4_ are located at the output of A_1_, A_2_, A_3_ and A_4_, respectively, as shown in Fig. 1. For each recombination of the input signals, the complex values of X_1_, X_2_, X_3_ and X_4_ are collected to form the 4 by 4 matrix *X* of Eq. (4). According to Eq. (5), the values of ${w}_{1}$, ${w}_{2}$, ${w}_{3}$ and ${w}_{4}$ of the 1 by 4 matrix ${W}^{out}$ can be obtained using *Y*^*target*^ and *X*. The coefficients of the terms *X*_*1*_, *X*_*2*_, *X*_*3*_ and *X*_*4*_ in Eq. (8) are equal to the 4 complex values ${w}_{1}$, ${w}_{2}$, ${w}_{3}$ and ${w}_{4}$ of ${W}^{out}$ forming 4 simultaneous equations. The phases of the complex numbers ${w}_{1}$, ${w}_{2}$, ${w}_{3}$ and ${w}_{4}$ are denoted by ${\theta }_{1}$, ${\theta }_{2}$, ${\theta }_{3}$ and ${\theta }_{4}$. The boundary conditions for ${\alpha }_{1}$, ${\alpha }_{2}$, ${\alpha }_{3}$, are real numbers between 0 and 1. By solving the simultaneous equations, ${\alpha }_{1}$, ${\alpha }_{2}$, ${\alpha }_{3}$, ${\varnothing }_{1}$, ${\varnothing }_{2}$ and ${\varnothing }_{3}$ can be obtained by

9 $${\alpha }_{1}=\frac{1}{1-{\left[{\frac{{w}_{1}}{{w}_{2}}e}^{i\left({\varnothing }_{1}\right)}\right]}^{2}}$$

10 $${\alpha }_{2}=\frac{1}{1-{\left[{\frac{{w}_{3}}{{w}_{4}}e}^{i\left({\varnothing }_{2}\right)}\right]}^{2}}$$

11 $${\alpha }_{3}=\frac{\left(1-{\alpha }_{1}\right){e}^{2i\left(-{\varnothing }_{2}-{\varnothing }_{3}\right)}}{\left(1-{\alpha }_{1}\right){e}^{2i\left(-{\varnothing }_{2}-{\varnothing }_{3}\right)}+{\left[\frac{{w}_{1}}{{w}_{4}}\right]}^{2}{\alpha }_{2}}$$

12 $${\varnothing }_{1}={\theta }_{2}-\frac{\pi }{2}$$

13 $${\varnothing }_{2}={\theta }_{4}-{\theta }_{3}-\frac{\pi }{2}$$

14 $${\varnothing }_{3}={\theta }_{3}-\frac{\pi }{2}$$

## Results and discussion

**Figure 2:**
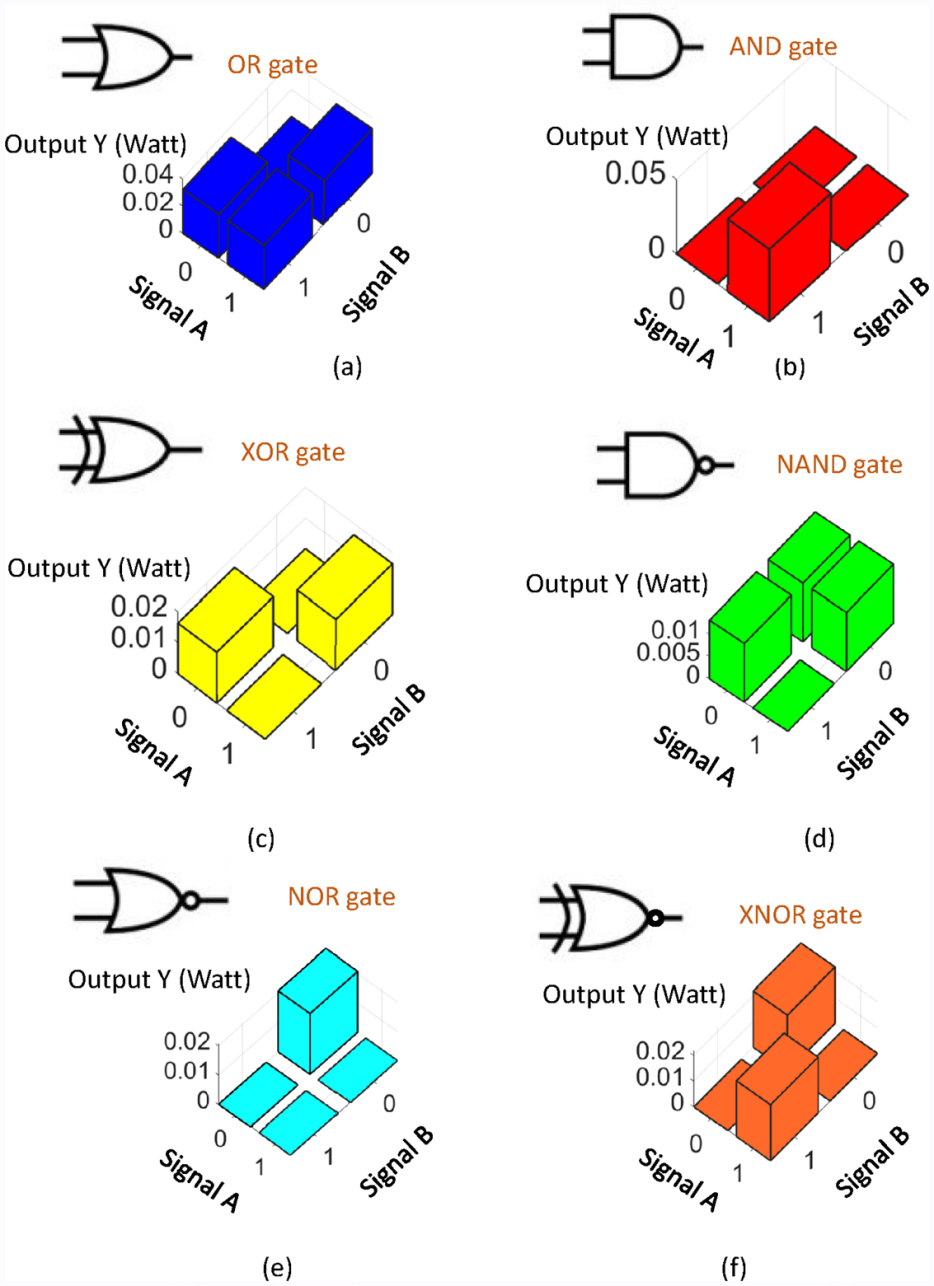
The output power of (**a**) OR, (**b**) AND, (**c**) XOR, (**d**) NAND, (**e**) NOR and (**f**) XNOR logic gates.

The ${Y}^{target}$ for the OR, AND, XOR, NAND, NOR, XNOR logic gates is [0 1 1 1], [0 0 0 1], [0 1 1 0], [1 1 1 0], [1 0 0 0] and [1 0 0 1], respectively. For each gate, the coupling ratios of the DC_A_, DC_B_ and DC_C_ in Fig. 1, α_A_, α_B_ and α_C_, are chosen randomly for 100 times to obtain the best tolerance. The detail calculation of tolerance will be discussed in the next section. The obtained α_A_, α_B_ and α_C_ for each gate are listed in Table 1. The corresponding coupling ratio of the directional couplers DC_1_, DC_2_, DC_3_ and the phase delay of the phase modulators PM_1_, PM_2_, PM_3_ calculated through Eqs. (2)–(14) are listed in Table 2. The output powers of the OR, AND, XOR, NAND, NOR and XNOR logic gates for different input signals A and B are shown in Fig. 2. For the electro-optical logic gates, the relation between the input signals and the output of the 2-layer MLP system follows the truth table of the corresponding logic gates. The reconfigurable logic gate can be achieved by tuning the parameters of the directional couplers^27–30^ and phase modulators^31^ as listed in Tables 1 and 2. It is worth mentioning that the phase delay of the phase modulators for NOR gate is close to null revealing that the phase modulators PM_1_, PM_2_ and PM_3_ might be removed. For the other gates, it is might be possible to find the solution to remove the phase modulators.

**Table 1:** The coupling ratio of the directional coupler DC_A_, DC_B_ and DC_C_.

	${\alpha }_{\mathrm{A}}$ (%)	${\alpha }_{\mathrm{B}}$ (%)	${\alpha }_{\mathrm{C}}$ (%)
OR	68	6	3
AND	73	89	98
XOR	56	83	96
NAND	22	8	7
NOR	58	23	81
XNOR	56	4	76

**Table 2:** The coupling ratio of the directional coupler DC_1_, DC_2_ and DC_3_ and the phase delay of the phase modulators PM_1_, PM_2_ and PM_3_.

	${\alpha }_{1}$ (%)	${\alpha }_{2}$ (%)	${\alpha }_{3}$ (%)	${\varnothing }_{1}$	${\varnothing }_{2}$	${\varnothing }_{3}$
OR	71	56	47	− 3.91°	− 11.43°	114.98°
AND	43	27	25	− 10.16°	− 20.43°	− 81.14°
XOR	**49**	37	64	**8.88°**	5.91°	47.83°
NAND	47	55	74	0.46°	5.82°	− 8.27°
NOR	51	48	1	0°	0°	0°
XNOR	57	52	25	7.14°	− 11.74°	106.32°

Significant values are in bold.

We use the visibility of the output signals of logic 0 and logic 1 to evaluate the tolerance of our logic gates. The definition of visibility is shown in Eq. (15)^32^

15 $$V=\frac{{I}_{min}^{1}-{I}_{max}^{0}}{{I}_{min}^{1}+{I}_{max}^{0}}$$

where ${I}_{min}^{1}$ and ${I}_{max}^{0}$ are the minimum light intensity of the high level (logic 1) and the maximum light intensity of the low level (logic 0), respectively. For example, for the output logic of XOR gate [0 1 1 0], if the output intensity of the 2-layer MLP system is [0.001, 0.013, 0.011, 0.002], we choose ${I}_{min}^{1}$ and ${I}_{max}^{0}$ as 0.011 and 0.002, respectively to calculate the visibility.

Since the coupling ratio of the directional couplers DC_1_, DC_2_, DC_3_ and the phase delay of the phase modulators PM_1_, PM_2_, PM_3_ are solved using Eqs. (2)–(14), the light intensity of the low level (logic 0) is null. The corresponding visibility of the output signals for all logic gates is unity.

During the fabrication of the directional couplers by optical fibers or by silicon waveguides, 1% error of the coupling ratio may happen. During the operation of the phase modulators by applying the voltage on the device, 1% error of the phase delay may occur. We calculate the visibility of the output signals as the coupling ratio of DC_1_, DC_2_, DC_3_ and the phase delay of the phase modulators PM_1_, PM_2_, PM_3_ are increased with 1% error. For example, for XOR gate, *α*_1_ obtained from Eq. (9) is 49% as listed in Table 2. As *α*_1_ is increased from 49 to 50% (*α*_A_, *α*_B_, *α*_C_, *α*_2_, *α*_3_, *ϕ*_1_, *ϕ*_2_ and *ϕ*_3_ are left unchanged as listed in Tables 1 and 2), the visibility of the output signal is calculated to be 0.99. For another example, for the XOR gate, as ϕ_1_ is increased from 8.88° to 12.48° (increasing 3.6° = 1% from – π to π), the visibility of the output signals is 0.91. For 100 calculations of different *α*_A_, *α*_B_, *α*_C_ of each gates, the highest visibility of the 2-layer MLP system for all logic gates due to 1% error of the devices is listed in Table 3.

**Table 3:** The visibility of the output signals for 1% error of coupling ratio of the directional couplers DC_1_, DC_2_, and DC_3_ as well as the phase delay of the phase modulators PM_1_, PM_2_, PM_3._

	${\alpha }_{1}$	${\alpha }_{2}$	${\alpha }_{3}$	${\varnothing }_{1}$	${\varnothing }_{2}$	${\varnothing }_{3}$
OR	1.00	0.99	1.00	0.98	0.94	1.00
AND	0.99	1.00	1.00	0.93	0.99	1.00
XOR	0.99	**0.98**	1.00	0.91	0.89	0.99
NAND	0.99	**0.98**	1.00	0.93	0.86	1.00
NOR	**0.98**	1.00	0.99	**0.83**	1.00	1.00
XNOR	0.99	1.00	1.00	0.89	0.94	1.00

Significant values are in bold.

We can observe that for 1% error of the coupling ratio of the directional couplers DC_1_, DC_2_ and DC_3_, the worst visibility is 0.98. For 1% error of the phase delay of the phase modulators PM_1_, PM_2_ and PM_3_, the worst visibility is 0.83. It seems that the results could be acceptable showing that the reconfigurable optical logic gates can be achieved even with the fabrication or operation errors. In electronics, a flip-flop or latch is a circuit that can be used to store state information with a pair of cross-coupled NOR of NAND gates. The further application of the reconfigurable optical logic gates is to build the flip-flop. The optical memory could be realized.

## Conclusion

In this work, we successfully establish an optical structure which can achieve EO logic gates. Thanks to this 2-layer-MLP structure, we can carry out the OR, AND, XOR, NAND, NOR and XNOR logic gates. In other words, we could take advantage of one optical neural network to achieve the most basic logic gates in optical approach. According to Eq. (5), we obtain the coupling ratio of the directional couplers DC_1_, DC_2_, and DC_3_ and the phase delay of the phase modulators PM_1_, PM_2_, PM_3_ under the ideal condition. The tolerance of the optical devices in our optical neural network is studied. The visibility changes of the output signals due to the 1% error during the fabrication and operation of the optical devices could be acceptable indicating that the reconfigurable optical logic gates can be realized in one optical configuration. Comparing our EO logic gates to the conventional electric logic gates, this EO logic gates have the capability of higher operating frequency, and high speed. In our study, the logic-gates functions among OR, AND, XOR, NAND, NOR and XNOR can be designed when the PMs and the coupling ratio of DCs are tuned in this EO logic gates. Thus, the control process is simpler. The present design uses the neural networks without recurrent property. The calculation performance could be faster than that of the previous work^5^. The optical logic gates can be applied to build an optical memory using the latch structure to store the information.

## Data Availability

The datasets used and/or analysed during the current study available from the corresponding author on reasonable request.
